# Infrared Sensor System for Mobile-Robot Positioning in Intelligent Spaces

**DOI:** 10.3390/s110505416

**Published:** 2011-05-18

**Authors:** Ernesto Martín Gorostiza, José Luis Lázaro Galilea, Franciso Javier Meca Meca, David Salido Monzú, Felipe Espinosa Zapata, Luis Pallarés Puerto

**Affiliations:** Department of Electronics, University of Alcalá, Alcalá de Henares, Madrid, 28801, Spain; E-Mails: lazaro@depeca.uah.es (J.L.L.); meca@depeca.uah.es (F.J.M.M.); david.salido@depeca.uah.es (D.S.M.); felipe@depeca.uah.es (F.E.Z.); luis.pallares@depeca.uah.es (L.P.P.)

**Keywords:** infrared position measurement, phase measurement, robots, sensors

## Abstract

The aim of this work was to position a Mobile Robot in an Intelligent Space, and this paper presents a sensorial system for measuring differential phase-shifts in a sinusoidally modulated infrared signal transmitted from the robot. Differential distances were obtained from these phase-shifts, and the position of the robot was estimated by hyperbolic trilateration. Due to the extremely severe trade-off between *SNR*, angle (coverage) and real-time response, a very accurate design and device selection was required to achieve good precision with wide coverage and acceptable robot speed. An *I/Q* demodulator was used to measure phases with one-stage synchronous demodulation to *DC*. A complete set of results from real measurements, both for distance and position estimations, is provided to demonstrate the validity of the system proposed, comparing it with other similar indoor positioning systems.

## 1. Introduction

An awareness of the context of this research, Mobile Robotics in Intelligent Spaces [1], is extremely important in order to appreciate the special requirements of this sensorial system. Although one might be tempted to compare this system with a conventional commercial infrared (*IR)* telemeter, the requirements differ enormously, as will be explained at the end of the introduction. Other concepts similar to Intelligent Space (*IS*) are emerging nowadays, and whilst their names (Pervasive Computing, Ambient Intelligence, Context-Aware Spaces, *etc.*) might be different, they all share the same philosophy: the environment (the Space) has the capacity to receive information, process it, take decisions and act on itself. The environment contains most of the complexity of the system, including sensors and networks, and most of the intelligence. All the aforementioned systems are human-centred, conceived to satisfy human needs in the environment, and generally employ cameras as the sensors, though some other types of sensor may also sometimes be used as a complementary source of information. Hashimoto proposed in 1999 the notion of Intelligent Space [2], in which intelligence is distributed within a defined space [3], where humans and robots share this (human-centred) space, and a sensorial system mainly based on cameras is used. Other, similar spaces currently exist, although not specifically termed Intelligent Spaces, with a human-centred design approach and again using cameras as the sensors. Given the context explained so far, the requirements for a local positioning system (*LPS*) are: (a) the robot must be as simple as possible, and this affects all the on-board electronics; and (b) the sensors placed in the environment must be simple and modular, in order to be easily scalable, easy to maintain and low cost, as the space will be provided with a high number of these elements. With regard to the sensorial system, its technical features must meet the following requirements: (1) acceptable precision; (2) coverage, that is, the emitter and receivers must have angles which are wide enough to enable them to see each other from different points within a reasonably large locating cell; and (3) real-time performance. The position of a mobile robot in an *IS* is detected by the Space itself, and several approaches have been proposed as local positioning systems (*LPS*) which are intended to work as a kind of indoor *GPS* [4], since *GPS* does not work properly indoors [5]. In *LPSs*, non-camera sensors are mainly based on ultrasound [6,7] and *RF* [8]. Very recent solutions have made use of existing *RF* wireless infrastructures such as Ultrawideband (*UWB*) or Radio Frequency Identification (*RFID*), or even the camera of a phone [9]. They typically work with signal-intensity levels [10] and currently have a precision range of around one meter [4,11]. The position can be obtained by using angle measurements (triangulation) [12] or distance measurements (trilateration) to fixed reference points placed in the environment. In the former case, although the position is relatively easy to solve it produces high error over long distances compared to the latter solution. In hyperbolic trilateration, the differences in distance to the reference points are obtained [13] rather than the direct distances to those points, so that all common offset-errors, including uncertainty in transmission-detection synchronism, are removed.

For trilateration, either distances or their differences must be obtained, and this can be achieved by measuring time of flight (*TOF*), or differential *TOF’s* [14]. Although time of flight is suitable for ultrasound (*US*), it is still not widely used in the case of *IR*, as a precision of picoseconds in time measurement is required in order to achieve a distance precision of 1 cm or below. Nevertheless, interesting studies reporting ps-*TOF* measurements for telemetry have been published in recent years [15,16]. As proposed in this paper, one alternative is to measure phase-shifts in order to obtain the distances to be used for positioning, employing an In-phase/Quadrature (*I/Q*) demodulating structure. Although the same structure is used in [17], this was completely digital and not aimed at *LPS* applications but rather for telemetry purposes. Furthermore, interesting undersampling techniques were used, and a 2-wavelength approach proposed, in [18]. As regards *IR* locating systems, most of them are based on cameras receiving signals from beacons, as in [19], or on a laser device [20], as in the *iGPS* discussed in [4]; however, in this case the system requires some kind of rotating device, rendering it more complicated and expensive. The use of *IR* may be of interest as many problems encountered with *US* are due to multipath and multi-echo interferences, and *RF* may also experience interferences between different rooms. For a distance-measurement precision of 1 cm, a very high precision in phase measurement is necessary (0.05°), considering a modulation frequency of 4 MHz; commercial *IR*-telemeters are able to provide this level or more of precision. It should be noted that a telemeter uses synchronism between emitted and received signals to carry out a directional measurement in which optics are used to focus the reflected power from the target on the detector, thus working with a high *SNR*. However, in an *LPS* there is a further requirement, as previously mentioned—Coverage—which implies that the emitter needs a wider emitting pattern so that three or four detectors (depending on its position) can see it simultaneously. However, the wider the emitting pattern is, the less power per solid-angle unit is obtained, hence less *SNR*, and consequently worse precision. Moreover, telemeters offer a precision range that improves as the measuring-time increases (longer integration-time), reaching a few seconds, but in this application there are real-time constraints that preclude such an increase in time. Therefore, it is more reasonable to compare the performance of this system with other *LPSs*, even though implemented with different technologies, rather than with a telemeter. A comparison between different *LPSs* is shown in the results section.

The aim of the study presented here was to develop an *LPS IR* sensorial system for implementation in an Intelligent Space, meeting the five conditions mentioned above and with a competitive level of precision. The design consisted of one emitter and a single photodetector for each receiver. Other, more complex solutions were discarded for all the reasons explained above. Developing solutions for *ISs* with different sensors is of great interest as their information can be merged. The system proposed represents a novel approach developed to fulfill the considerations discussed above.

## 2. Background

Several technological approaches to *LPS* systems are currently being developed in research fields related to robotics and intelligent spaces. These include rather different structures, both for position estimation and the measuring system, normally involving some kind of distance or differential distance estimation. The method used to measure these distances and to calculate a position from them characterizes and defines a specific *LPS*. Ultrasound-based systems, on which the Department of Electronics at the University of Alcalá has been working for several years [21,22], can be considered the most similar to the system proposed in this paper, in terms of the use of differential distances (in this case, calculated from *TOF* measurements) to fixed reference points formed by receivers, and position estimation by spherical or hyperbolic trilateration.

Most current positioning systems are based on a solid measuring technology, often using high performance commercial transducers to form their sensorial system, and focusing research efforts on the development of positioning algorithms and techniques. In this case, most of the complexity of the proposed *LPS* centers on the sensorial system used to measure differential distances, based on an optical phase-shift measurement through a non-guided direct link, since a sensor that would be capable of providing the *SNR*, coverage and time stability needed is not currently commercially available. Thus, it was necessary to develop the entire sensorial system, in order to obtain a high performance *IR* sensor that would meet the given specifications, and the design and physical implementation of this was made possible thanks to this department’s background in the related fields of radiometry, optoelectronics and applied optics.

## 3. The LPS Sensorial System

The concept of an *IR-LPS* based on differential phase-shifts, together with other aspects of the system, have already been discussed in [23]. In [24], guidelines for the measuring system were addressed, where the term *DPOA* (differential phase-shift-of-arrival), analogous to the classical term *DTOA* (differential time-of-arrival) in *TOF*-based systems, was defined.

The main idea of the system is to locate a robot by measuring differential distances from it to determined reference points, which are, in turn, the detectors. Differential distances are directly calculated from the measured phase-shifts and introduced into a hyperbolic trilateration (*HT*) non-linear equation system, to obtain the robot location [25]; an explanation is given in [26] of the basics of triangulation equations and a linearized formulation applied to wireless *RF*-location. In *HT*, every measurement defines a hyperboloid which is the loci of possible locations for the robot. The solution is found with several differential measurements so that, ideally, the robot position would be located in the intersection of hyperboloids. In practice, the *HT*-equation system is solved by a non-linear-least-squares estimation plus a further numerical solution using the Gauss-Newton iterative method. This is a commonly accepted strategy to solve the aforementioned *HT* nonlinear system [27]. The precision of a particular location can be characterized by two standard deviations, σ_1_ and σ_2_, corresponding to the major and minor axis of an ellipse of around 95% location-probability (2σ_1_ and 2σ_2_) [28].

The main difficulty in developing the described system lies in the sensors, since working with a frequency above 1 MHz strictly limited the maximum working power of the photonic devices. It was therefore necessary to develop a sensor capable of providing, from very low received optical power, a sufficiently high *SNR* signal as to measure distances that lead to a positioning precision under 10 cm.

**Figure 1.**
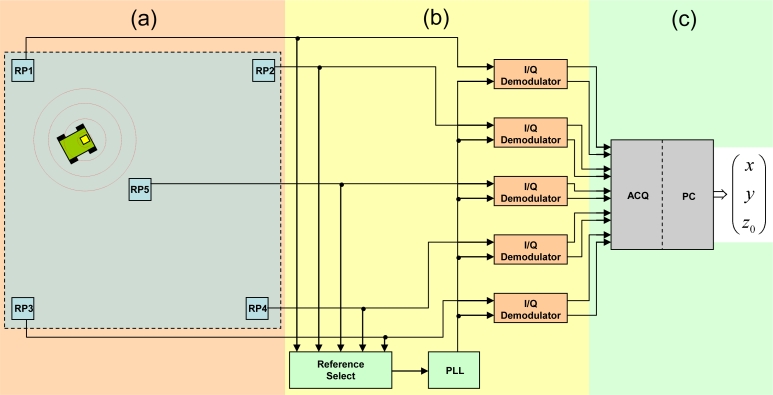
System block diagram.

A schematic representation of the entire system is given in Figure 1. Figure 1(a) represents the emitter installed on the mobile robot and the sensors covering the positioning area; Figure 1(b) represents the signal treatment block, containing the reference signal recovery and *I/Q* demodulation stages. Finally; Figure 1(c) depicts the acquisition (*ACQ*) and digital processing stages undertaken on a *PC*, where distances are calculated and the resulting non-linear system is solved to obtain the estimated position. The *Z* coordinate, corresponding to the height of the emitter boarded on the robot, is known beforehand and not provided by the positioning system; the subscript 0 is used to point this out.

**Figure 2.**
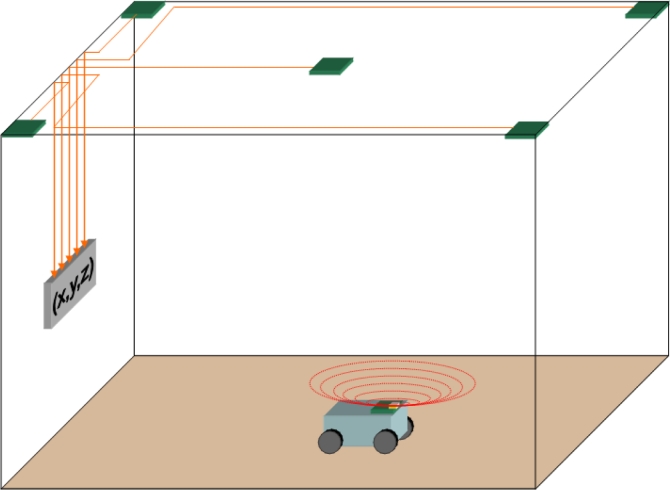
Basic locating cell.

An intensity modulated *IR* signal is sent from a high power emitter installed on the robot and is received by five sensors, *RP_1_* to *RP_5_*, placed in the center and at every corner of the upper plane of a cubic cell, as shown in Figure 2. The highest amplitude signal received is sent to a *PLL* to generate a reference signal, the phase shift of which, with respect to each of the other received signals, is measured. This is carried out by *I/Q* demodulators whose outputs, digitalized into a *PC*, comprise the data used for position estimation by *HT*.

The frequency used for the *IR* modulation was 4 MHz, chosen as a trade-off between sensitivity and correct component behavior. As regards precision in the phase-shift measurement, the higher the frequency, the higher the precision for the corresponding distance, but at the same time, some devices may not operate properly. This is related to the natural trade-off between power and working frequency, mainly affecting the emitter circuitry, being the one working with the highest power levels of the system. To illustrate this, note that the current source driving the *IRED* should provide an rms current over 0.5 A modulated at 4 MHz, which is a rather high power-frequency relation for any up-to-date device bandwidth.

## 4. LPS-Parameters Trade-Off

**Figure 3.**
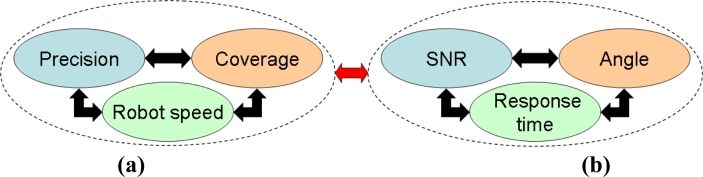
**(a)** System-level trade-offs; **(b)** Electronic-level trade-offs.

As previously explained, a *LPS* must meet the three main requirements (precision, coverage and speed) satisfactorily in order to provide an adequate solution for mobile robotics, that is, positioning a robot within an acceptable error threshold, over a sufficiently large area and providing the position information with sufficient speed to track a robot moving at certain speed. These are, by definition, competing parameters, which translates into a strong trade-off between device selection and design of the different stages of the *LPS*. Figure 3 shows these interrelationships, together with the corresponding parameter at the electronic-level design of the system. As can be seen, precision is directly related to achieving effective *SNR* levels in the signals from which distance information will be extracted, coverage is directly dependant on the working angles of both emitting and receiving devices, and speed is defined by response time of devices and electronics as well as by position estimation processing time.

Achieving higher *SNR* levels usually involves two different approaches; reducing noise in the electronics by means of adequate design and device selection, and improving the efficiency of the optical link in order to obtain higher signal levels. From the point of view of the emitter, the second approach implies concentrating emission over a smaller solid-angle, reducing the coverage accordingly, and, from the point of view of the receiver, widening the reception area, which can be achieved either by using a larger device, increasing the shunt capacitance of the photodiode and thus raising its response time, or by using an optical system, which, considering the device sensitive area, reduces the reception angle and consequently, the coverage.

On the other hand, in addition to requiring faster devices, raising system speed is strongly related to the defined bandwidth after demodulation, as well as to the processing time, since in general, a slower system allows narrower bandwidth and hence higher *SNR*, in addition to longer integration time to compensate dispersion.

The relationships between these three main parameters, from a high-level standpoint, are reflected both in the device selection and circuit design of every stage of the system. These low-level trade-offs will be explained below.

### 4.1. Emission

Given the specific conditions of the *LPS*, the emitter should provide the best possible performance regarding competing parameters, namely, emitted power per solid angle unit, angular width and maximum working frequency. Whilst very high performance devices are available on the market, generally, when one of their features is outstanding, and the rest are strongly penalized. This might be of little significance, depending on the application; for example, the most accurate systems in telemetry are based on *LASER* emitters which can concentrate all the radiation in a small area due to measuring in a straight line and having a relatively high response time. In the case of the *LPS*, it was absolutely necessary for the chosen device to be sufficiently good at the three aforementioned parameters, which implies a high trade-off between them.

As is usual in electronic devices, there is an inverse relationship between power and response time, narrowing selection of the emitter device to those that can operate above 4 MHz. On the other hand, to maximize system coverage, the emitted optical power should be spread over the largest possible solid angle; there is no direct dependence between emitted power and angular width in the device itself, but this coverage condition prevents the use of any optical system to concentrate the emitted power over a smaller solid angle, which would increase the power reaching a specific detector but decrease the number of detectors simultaneously seeing the emitter. Minimum acceptable device performance for the proposed system would be over 1 W emitted power with at least a 60° half-angle; features which are not readily available in current fast response devices.

### 4.2. Reception

The sensor receiving the signal coming from the *IR* transmitter is composed of a receiver device and signal conditioning electronics. As in the case of the emitter, selection of the receiver device must be carried out considering the trade-off between conflicting parameters: active area, working frequency and field of view (*FOV*). There is a strong trade-off between active area and maximum working frequency in typical *IR* detector devices such as photodiodes and phototransistors; this is due to junction capacitances increasing as active area increases, creating a low-pass filtering effect that limits the working frequency.

Another problem regarding the use of optical systems also emerges in receiver devices; whilst it is possible to virtually widen the active area of the detector by means of an optical system, this reduces the viewing angle accordingly, establishing a new trade-off between received power and *FOV*. The final design did not employ an optical system because the rise in optical gain would have implied an unacceptable narrowing of the viewing angle for the current detector area, and higher mechanical complexity of the sensor, with the consequent maintenance problems; note that an *IS* is formed by a high number of sensors so the probability of a failure is closely related to their complexity.

All the devices forming an *LPS* constitute key factors in its final performance; nevertheless, the receiving stage, which may at first seem simple since it comprises a classical design formed by an *I/V* conversion stage plus amplifying and filtering, is of extreme importance. This is the noisiest stage in the whole system; the final *SNR* of the system depends directly on the noise at this stage and the bandwidth of the signal-treatment block output filter. Therefore, a design focused on reducing noise addition and component value dependence was required, and special care was taken when selecting both active and passive devices integrating the receivers.

As is usual in electronic measuring systems, it was necessary during the design of the conditioning stage to pay particular attention to minimizing noise addition to the received signal, and this was especially complex in the design proposed here due to the very high gain (some tens of kΩ) needed in order for the transimpedance amplifier to produce an adequate voltage level from the generated photocurrent to make the most of the dynamic range of subsequent stages. On the other hand, equal importance had to be given to stability, specifically focusing on minimizing propagation time uncertainty due to component value variations; this condition implied a sensitivity study that added constraints to the filtering stages, causing a trade-off between stability and *SNR* improvement in the conditioning stage.

### 4.3. Signal Treatment

The proposed signal treatment block is basically composed of an *I/Q* demodulator. Each received signal was multiplied by quadrature references of its frequency, recovered by means of a PLL, shifting the received information down to two *DC* signals in sine and cosine form where phase could be extracted. The demodulator outputs were low-pass filtered before digitization to eliminate the high frequency components resulting from the products and to reduce noise bandwidth. The main trade-off related to this stage lay in the low-pass filtering of the DC components, which critically defined the final relationship between precision and mobile robot maximum speed. Strong filtering will highly reduce dispersion, hence improving precision in static measurements, but the more restrictive the filtering, the higher the error will be when tracking varying signals when the robot moves.

Although the demodulator itself did not introduce a strong limitation on any of the three main parameters under study, it is important to note that correct modeling of its behavior, and the subsequent fixed errors correction process in the estimation [29], were absolutely necessary to achieve acceptable accuracy in the final position.

## 5. Proposed Solutions

Having now explained and specified the main parameter trade-offs for the different system stages, in this section the criteria for the device selection and the electronic design of every subsystem are discussed.

### 5.1. Emitter

The device selected for the system was the *IRED SFH4231*, manufactured by Osram, which maintains an excellent balance between all the parameters; in fact, it is one of the best devices currently available on the market for the required application. It has high optical power, a wide angle and low response time, and represents an extremely up-to-date and suitable choice as can be seen in Table 1, where the main features of some *IREDs* considered for the prosed application are compared, together with a qualitative evaluation of their main advantages and disadvantages. These features are: maximum emitted power (P_max_), maximum emitted radiant intensity (I_max_), 3dB-bandwidth (f_max_), half-power angle (**θ_1/2_**), peak wavelength (**λ_p_**), maximum direct current in continuous and pulsed emission (I_Dmax_).

**Figure 4.**
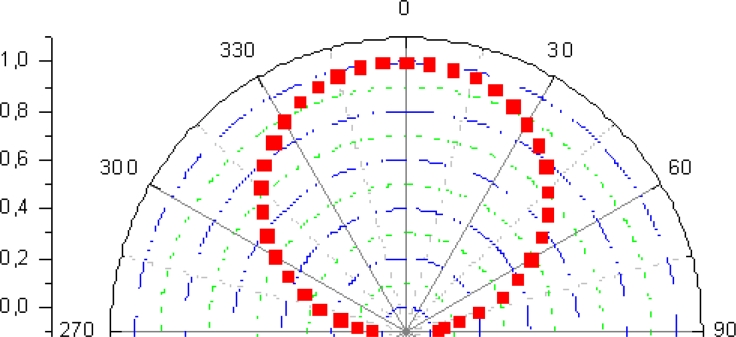
Radiation pattern of the emitter device.

Figure 4 shows the normalized radiation-pattern of the *IRED* obtained in our tests. As can be seen, its half-power emitting angle was around ±60°, which matches the manufacturer specifications. This is a reasonably satisfactory emitting angle to work with.

**Figure 5.**
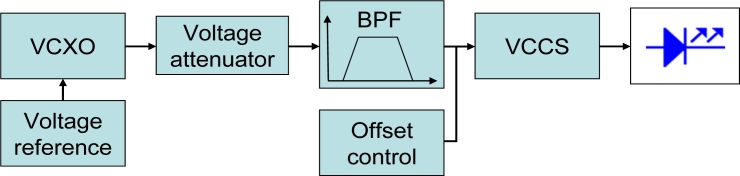
Emitter circuit.

The bias circuit of the emitter is shown in Figure 5. A voltage controlled crystal oscillator (*VCXO*) generates a 4 MHz tone; this signal is attenuated to an adequate level to make the most of the led’s dynamic range and band-pass filtered. A *DC* component, generated from a voltage reference, is added to the resulting sine signal to drive a voltage controlled current source (*VCCS*) formed by an n-channel power *MOSFET* transistor (PD55015) and a low-noise fast *FET* operational amplifier (*OA*) (AD8065). This current supplies the *IRED* with the adequate bias level. With this circuit, the *LED* is driven by a pure sine-signal, at a current bias point of 475 mA and a peak current value of 900 mA. Assuming a linear emissivity of 0.5 W/A in the current range used for polarization, and a negligible response time for the working frequency (both data obtained from the device datasheet), the optical emission results a sinusoidal-intensity-modulated signal with 400 mW peak to peak power value.

### 5.2. Receiver

**Figure 6.**
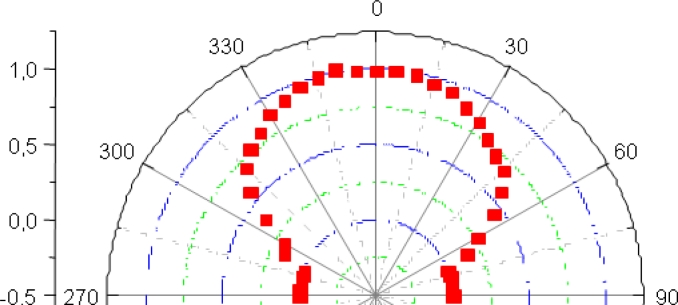
Reception pattern of the receiver device.

The device chosen for the application was the photodiode *SD100-11-31-221* manufactured by *API*. Figure 6 shows the normalized reception-pattern of the photodiode obtained in the developed tests. A high responsivity level of up to ±60° can be observed, as specified in the device documentation.

**Figure 7.**
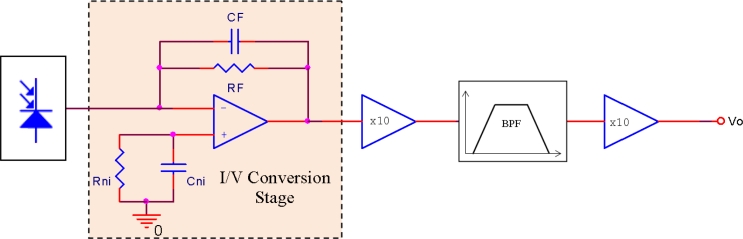
Receiving circuit.

The receiving circuit is shown in Figure 7. As can be seen, it is formed by the receiving photodiode, an *I/V* conversion stage carried out with an *OA*, an amplifying stage divided into two sub-stages due to bandwidth requirements, and a passive, wideband, second order band-pass filter.

**Figure 8.**
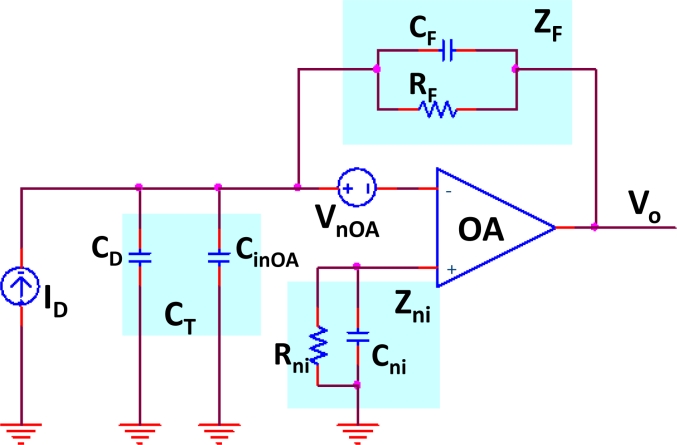
I/V converter equivalent circuit.

The *OA* chosen provided ultra-low offset current, offset voltage and noise voltage. The transimpedance resistor was very low tolerance and the system pole was placed using the *R* and *C* parameters at exactly 4 MHz in order to attain maximum stability in propagation time. The *SNR* at the output of the *I/V* conversion stage was maximized, but constrained to the mentioned design condition of maximum time-delay stability against any changes in transimpedance feedback passive components (resistance and shunt capacitor). To obtain the noise transfer function (*NTF*), the equivalent circuit to the one in Figure 7 was used, whose schematic is shown in Figure 8.

In this circuit, *I_D_* is the photodiode current and *C_D_* is its shunt capacitance; the effect of the resistances in the diode model was negligible. The capacitance C_T_ is the equivalent capacitance formed by *C_D_* and *OA* input capacitance, C_inOA_. The total equivalent noise-voltage referred to the input is represented by *V_nAO_*. The *I/V* conversion impedance *Z_F_* is formed by *C_F_* and *R_F_*, the latter tuning the cut-off frequency of the circuit. Z_ni_ compensates for the effect of bias currents through R_F_, and C_ni_ is set to reject the voltage noise generated by the *OA* input noise current through R_ni_. Thus, given the frequency-dependant transimpedance transfer function of the circuit in Figure 8, *Z(jw)*, the output voltage signal, *V_o_*, in the frequency domain, is:

(1) $$v_{o}(jw)=i_{D}\cdot Z(jw)=i_{D}\cdot A_{o}(jw)\frac{A_{F}(jw)}{1+jwA_{F}(jw)C_{e}(jw)}$$

with:

(2) $$A_{o}(jw)=\frac{A_{vo}}{1+jw/w_{cH}};\;\;A_{F}(jw)=\frac{R_{F}}{1+A_{0}(jw)};\;\;C_{e}=C_{T}+C_{F}[1+A_{o}(jw)];\;\;\;C_{T}=C_{inAO}+C_{D}$$

where *A_o_(jw)* is the open-loop operational-amplifier voltage-gain, with *DC* gain *A_vo_*, and cutoff-frequency *w_cH_*. *C_T_* is the total input shunt capacitance *C_inAo_* and *C_D_*. The non-ideal effects modified the ideal transimpedance response, but the upper cutoff-frequency remained tuned by the transimpedance *Z_F_*. On the other hand, the noise power related to the output *N_RTO_* was obtained from the circuit in Figure 8. *N_RTO_* was calculated considering both voltage (V_nOA_) and current noise (i_nOA_) of the *OA*, thermal noise of *R_F_* (*4K_B_T/R_F_*, where KB is the Boltzmann constant and *T* the temperature in *K*), and current noise of the photodiode (*I_DN_)*, all factors weighted by the corresponding transfer function effect:

(3) $$N_{0i,f}=\sum_{p}n_{ip}^{2}={\left(V_{nAO}\right)}^{2}\frac{1+{\left(2\pi fR_{F}(C_{F}+C_{T})\right)}^{2}}{1+{\left(2\pi fC_{F}R_{F}\right)}^{2}}+\left(\frac{4K_{B}T}{R_{F}}+I_{DN}^{2}+i_{nAO}^{2}\right)\frac{R_{F}^{2}}{1+{\left(2\pi fC_{F}R_{F}\right)}^{2}}$$

**Figure 9.**
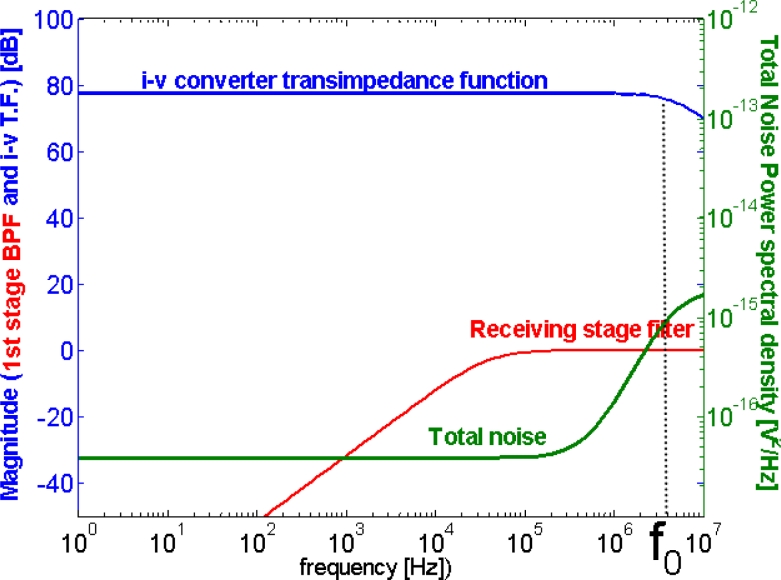
Frequency domain plots: sensor transimpedance function, filter transfer function (only lower cutoff frequency is shown) and noise power spectrum.

The plot in Figure 9 depicts the transimpedance transfer function appearing in (1), the band pass filter and the noise transfer function (*NTF*) defined by (3), for the actual values of the components in the design. As can be seen on the right-side axis, there is a 10^−l5^ V^2^/Hz noise power-density value at 4 MHz, optimized as explained in previous paragraphs. However, at the point *V_o_* in Figure 7, the total noise power resulting from integration in the wide pass-band is much higher than the signal power received from many of the locations in the positioning cell, reaching a −20 dB *SNR*, as will be discussed in subsequent sections.

Note that should any single parameter of any component change, either that of the emitter or of the sensor, both the *NTF* and the signal-*TF* would change. This would cause a change in the final noise-power level, hence in the effective *SNR*, and finally, in the precision of the measurement.

### 5.3. Non-Guided Link

The link between transmitter and receiver was a wireless infrared *LOS* (line of sight) link, where the interfering contributions from reflections remain within the desired precision margin. This can be guaranteed by maintaining a dead zone of about 40 cm between the robot and the walls [29,30]. The expression for the electrical current rms-value generated at one photodetector [29] is:

(4) $$\begin{array}{l} I_{0i}=𝔕\cdot {\left(\frac{P_{emx}A_{S}}{D_{i}^{2}}\text{cos}(\theta_{ei})\text{cos}(\theta_{ri})\Pi \left(\frac{\theta_{ri}}{\left(FOV/2\right)}\right)\right)}^{2}=𝔕\cdot {\left(\frac{P_{emx}A_{S}}{D_{i}^{2}}{\text{cos}}^{2}(\theta )\Pi \left(\frac{\theta }{\left(FOV/2\right)}\right)\right)}^{2}\\ \text{where}\;\;\;\Pi \left(\frac{\theta }{W}\right)=\{\begin{array}{ll} 1 & if\;\theta <W\\ 0 & if\;\theta >W \end{array} \end{array}$$

where the optical intensity at each receiver, *I_0i_*, is the photodiode current *i_D_* appearing in (1). 𝔕 represents the photodiode responsivity, *P_emx_* is the optical power per solid angle emitted in the normal direction, *A_S_* is the photodiode sensible area, *D_i_* is the Euclidean distance between emitter and receiver and *θ_ei_* and *θ_ri_* are the angles of the *LOS* referred to the emitter and receiver normal directions respectively. In the second part of the equation these angles are grouped into *θ* because of being equal thanks to the vertical orientation of both emitter and receiver maximum performance directions. Finally Π(*θ/W*) is a window function modelling the receiver’s field of view (*FOV*) mechanical limitation that causes a complete loss of signal from certain reception angle defined by *W*.

All the elements contributing to the *IR* link, including the emitter, the entire sensor and the wireless *LOS* link have thus far been presented. In Table 2, real voltage, noise and *SNR* levels are shown, in order to give an insight into actual working conditions. In the proposed locating cell, the best *SNR*-case was obtained at the minimum distance between emitter and receiver, 2.5 m, and the worst at a distance of 3.5 m. The signal amplitude was that given by the peak value of the module of *v_o_(jw)* in (1) at 4 MHz, and its measured value is shown in the table for the above-mentioned best and worst cases. The noise-power would be the result of integrating the spectral density power given by the *NTF* in all frequencies in the pass-band range, which is equal to the noise variance σ*_N_*^2^, computed from the real-time domain measurements.

**Figure 10.**
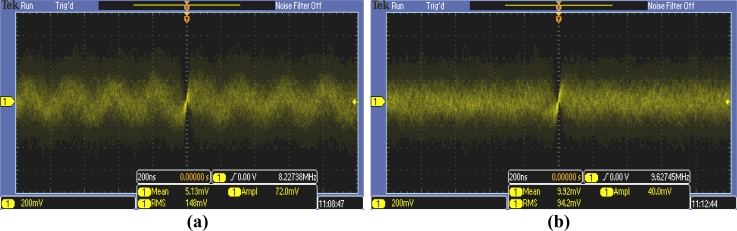
Signals in the output of the receiver: **(a)** Best case: (2.5 m, 0°); **(b)** Worst case: (3.5 m, 45°).

In Table 2, the results are shown of the tests to obtain signal, noise, and *SNR* levels at the sensor output. Meanwhile, Figure 10 depicts the voltage-signals at that point, for the best and worst cases. As can be seen in the table, although the sensor was carefully designed, the *SNR* reached very low levels due to the unfavorable conditions in angle and distance. Note that the angle related to measuring conditions affected both the emitter and receiver, *i.e.*, both devices were working at 45°. The measuring conditions were highly unfavorable, as can also be seen from the signals in Figure 10, and it is only thanks to the careful design of the sensor for optimizing the *SNR*, together with an accurate signal treatment block design, that the information is still usable.

The first two rows show data corresponding to the previously defined best and worst cases for the current test locating cell. The third row, not corresponding to any position inside this cell, is included to provide an idea of the strong trade-off between coverage and precision.

The output-signal of every sensor was fed into a signal treatment block, explained in the next section, where the effective *SNR* level will be defined.

### 5.4. Signal Treatment Block

**Figure 11.**
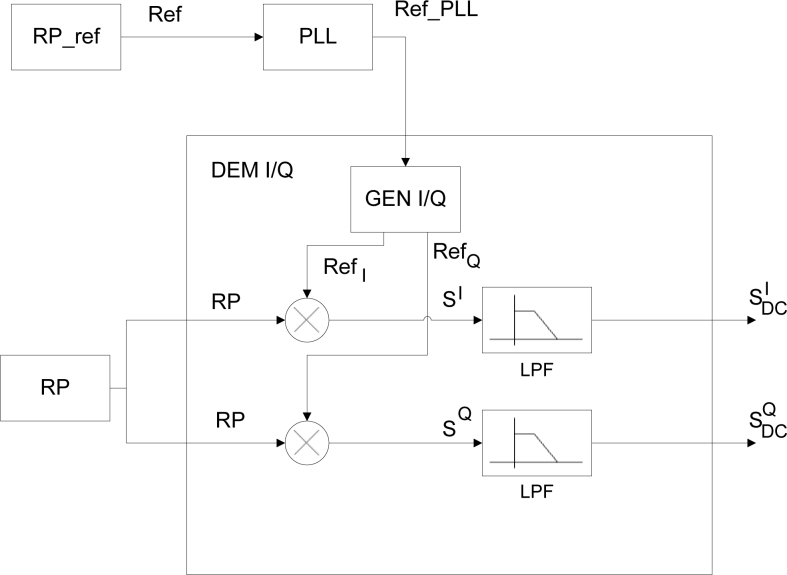
Signal treatment block.

Figure 11 shows the signal treatment block. Each sensor output voltage-signal, *RP*, was sent to an *I/Q* demodulator, together with the signal coming from the reference receiver, *Ref*, recovered by a *PLL*.

In the proposed application, the function of the *I/Q* demodulator was to extract the phase information from a sinusoidal signal and to output it in two *DC* signals. The reference signal, *Ref_PLL*, was used to generate the in-phase and quadrature signals, *Ref_I_* and *Ref_Q_* in Figure 11, to be multiplied with the input, *RP*, for *I/Q* demodulation. After low-pass filtering both multiplier outputs, the two resulting *DC* signals, *S^I^_DC_*, *S^Q^_DC_*, are:

(5) $$\begin{array}{l} S_{DC}^{I}=A_{0}\;\text{sin}(\phi +\phi_{c}^{I})+n_{v}^{I}\\ S_{DC}^{Q}=A_{0}\text{cos}(\phi +\phi_{c}^{Q})+n_{v}^{Q} \end{array}$$

where A_0_ represents the product of both *RP* and *Ref* plus internal gains of the demodulator, *ϕ* is the phase-shift between *RP* and *Ref* to be measured, $\phi_{C}^{I}$ and $\phi_{C}^{Q}$ are the fixed phase-errors of the entire system, corrected in the calibration process, and $n_{v}^{I}/n_{v}^{Q}$ are additive noise contributions (considered Gaussian) present in measurements of the demodulator outputs; note that they are considered different since they follow two independent physical paths, but their noise power is the same, and thus they have the same standard deviation. From the signals in (5), following the calibration and error correction process [29], arctangent estimation was used to obtain the phase-shift from which the final differential distance was calculated.

**Figure 12.**
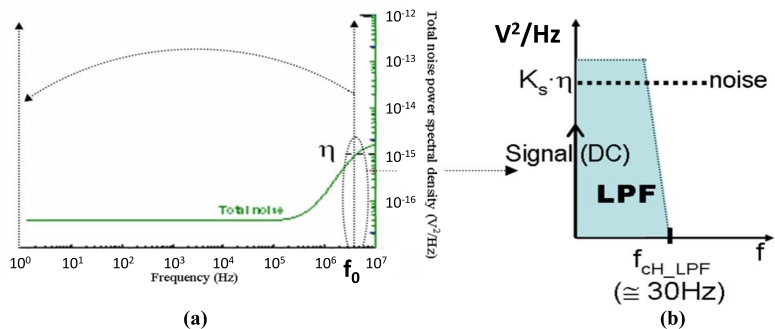
**(a)** Noise frequency-shift due to demodulation; **(b)** Final (low-pass filtered) total noise.

After demodulation, signal and noise spectra were shifted down an *f_0_* (4 MHz) frequency-step. As schematically shown in Figure 12, the final effective noise was the result of integrating the spectra into the output filter bandwidth, which is an active first order low-pass filter with a very narrow band (set to 30 Hz). Thus, only the part of the noise density power contained in that band, on the right-hand side of f_0_, contributed to the final effective noise. The value of the noise density-power at f_0,_ η, (see Figure 12(a)), can be considered constant in the band of the output low pass filter, *LPF* (see Figure 12(b)). The *LPF* was extremely narrow in order to identify substantial variations. Thus, in the context of this noise-band integration it can be considered flat noise; the constant *Ks* takes into account the multiplying constants in the circuit from sensor to output.

Table 3 shows the noise and *SNR* levels at different points in the circuit. It can be seen how *SNR* improved at the demodulator outputs with respect to the output at the sensor. The measuring conditions refer to the distance between emitter and receiver and both working angles. The amplitude used for the *SNR* calculation was always carried out at the sensor output; this was necessary because the information was transferred into a *DC* component in the demodulator, and an effective *SNR* level was always defined using this amplitude and the noise level in every point, providing a sound definition since the information power, as previously explained, was shifted down to *DC* but not altered. This is, the signal treatment block aims at reducing noise bandwidth while not modifying signal power, shifting the signal to *DC* allows the bandwidth reduction to be maximized.

Noise values were computed from real measurements taken at different points of the reception and signal treatment stages. The reduction of total noise is clearly illustrated in Table 3, which shows an increase of 26 dB between sensor and demodulator outputs. Following that, the ADC anti-aliasing filter caused remaining high frequency noise to be rejected and a final digital low-pass filtering, implemented in the *PC*, established the effective *SNR*, with a highly satisfactory improvement of 37.5 dB over the initial value thanks to the signal treatment block.

## 6. Results and Discussion

**Figure 13.**
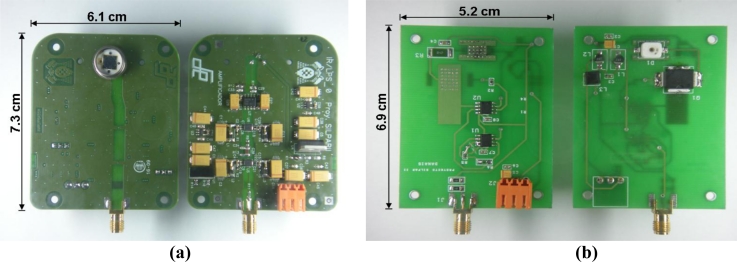
**(a)** Receiver PCB; **(b)** Emitter PCB.

Having explained the definitive designs adopted for every stage of the system and the resulting *SNR* levels defined for actual working conditions, in this section the results for distance and position measurements, together with a comparative chart of other indoor positioning systems, are presented. Before focusing on numerical results, the final emitter and receiver designs in *PCB* are shown in Figure 13.

**Figure 14.**
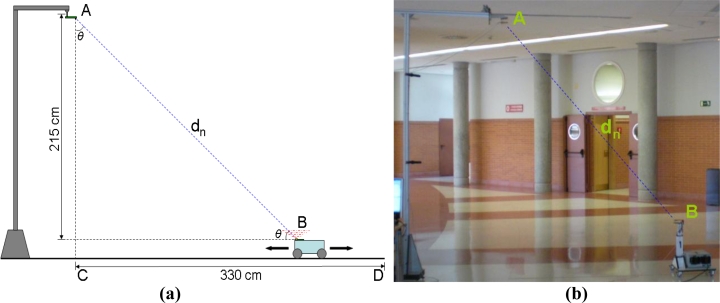
Setup for distance measuring using one receiver. **(a)** Setup diagram; **(b)** Actual setup.

Figure 14 depicts the setup used for distance measuring tests. The receiver was placed in position A, orientated so that its direction of maximum responsivity pointed vertically at the floor. The emitter was placed in position B, onboard a mobile robot at 60 cm above the floor so that its distance to the horizontal receiver plane was 215 cm.

The robot was moved through a set of positions between C and D in the diagram, that is, a position directly under the receiver (C), where measured distance should be 215 cm, and a position 330 cm away horizontally (D), where measured distance should be 394 cm. The set of positions was calculated so that distance increments in the diagonal were constant and equal to 20 cm. This setup covered the entire range of possible distances and angles the system should be able to work with according to previously defined performance specifications.

**Figure 15.**
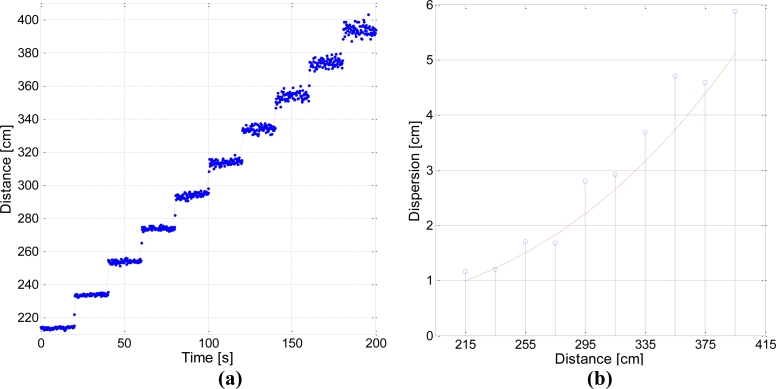
**(a)** Distance measurements using one receiver; **(b)** Dispersion for each distance (2σ).

Using this configuration, captures were taken for every position, averaging the data every 250 ms to obtain the resulting distances shown in Figure 15(a), where the blue dots represent estimated distance and the red lines represent the true value for every position. Figure 15(b) shows the dispersion measured for every position, defined as two standard deviations. The red line represents a radiometric simulation of the expected dispersion increase. Note that when the robot was moved away from the receiver, there was a high power reduction due to the distance increase, but also to the variation in both *θ* angles, hence the rapid dispersion increase shown in Figure 15.

It can be observed that dispersion for the worst case remained around ±6 cm, below 1.5% of the measured distance, and rather lower for positions closer to the receiver. As mentioned in the Introduction, these results may seem poor compared to current distance measuring systems, such as laser telemeters, which can achieve precision rates of under 1 mm for much longer distances, but it should not be forgotten that the sensorial system forming a LPS must meet two further critical requirements in addition to precision: coverage and response time, the trade-off of which with precision has already been explained in Section 4; coverage does not affect telemetry systems at all, whilst response time has a much less restrictive effect than in the case of an LPS.

Having defined distance measuring behavior, position estimation results will be shown, enabling the drawing of conclusions about performance of the entire system as an *LPS*. As mentioned in Section 3, once differential distances had been measured, position was estimated by hyperbolic trilateration. In this case, once the height of the emitter and the sign of the distance difference are known, only two hyperboloid equations are necessary to solve a position, that is to say, only two differential distances obtained from two receivers plus a reference distance are needed. Each new receiver added to the system introduces a new equation, the effect of which on dispersion in the final position estimation can be positive or negative, depending on the new differential distance SNR. Analyzing the resulting dispersion in distance measurements with one receiver and taking into account an SNR increase by a factor of $\sqrt{2}$ (considering that their respective noises are uncorrelated) when calculating differential distances with two receivers, it was concluded that the use of five receivers to cover all the positions in the cell was counterproductive, since they generated higher dispersion in the estimated position than the dispersion obtained using four receivers. Accordingly, the positioning cell was divided into four quadrants, where the furthest receiver was not taken into account.

**Figure 16.**
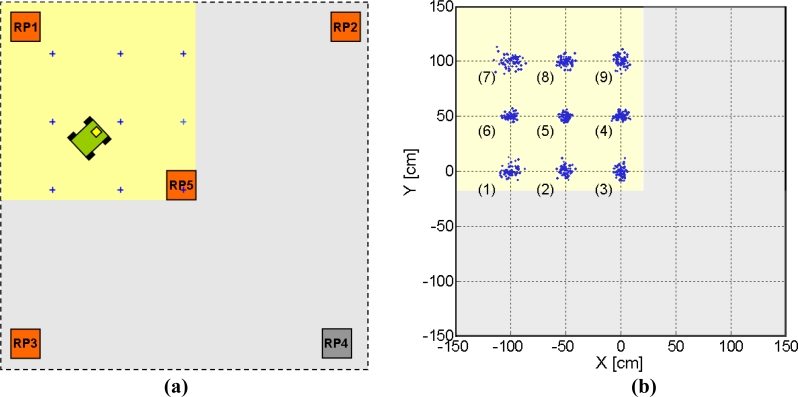
**(a)** Setup for position estimation; **(b)** Position estimation results.

One of these quadrants can be observed in Figure 16(a), where the active receivers are highlighted and the positions where the robot will be placed are marked with crosses. Figure 16(b) shows the results for test points (1) to (9), and true values are placed at the intersections of grid lines. The corresponding clouds of points represent the estimated positions.

**Figure 17.**
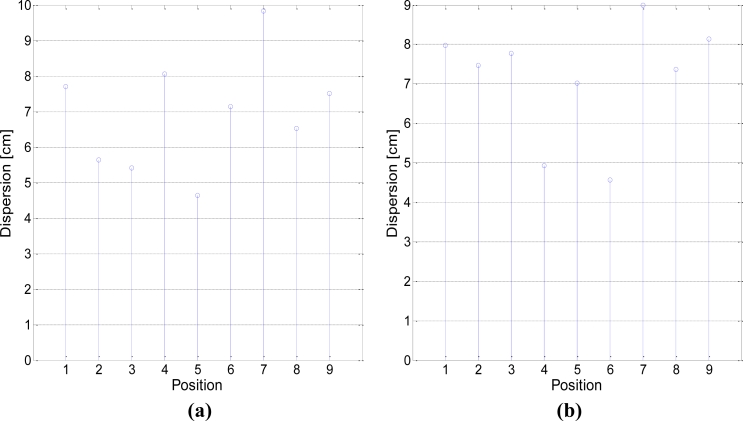
Dispersion for each position (2σ). **(a)** X axis; **(b)** Y axis.

Dispersions on both axes for position estimations are shown in Figure 17, calculated as two standard deviations. The position numbers correspond to the ones in Figure 16(b). It can be observed that dispersion remained below 10 and 9 cm for the x and y axes, respectively, with an average value of 7.3 cm in both directions. The differences in the dispersion levels between different points are due to the system geometry. As shown in distance results, *SNR* levels in every receiver keep a high dependence with position; this, together with the effect of the chosen reference, whose *SNR* affects all differential distances, determines the spatial dependence of precision. Nevertheless, this dispersion differences always remained below 5 cm. Note that these results are given for specific conditions of coverage (3 × 3 m^2^ covered with 5 receivers) and position data rate (250 ms); should any of these limitations be reduced, precision would consequently increase.

In order to provide an intuitive idea of the proposed system’s performance, a chart comparing this system with other state-of-the-art local positioning systems currently under research is presented in Table 4. Information about precision, signal used, and most usual measuring techniques is provided. The last two rows represent the system proposed in this paper, the first one corresponding to actual results with current devices and the second one to estimated results assuming an improvement in photonic devices performance.

Systems based on *RF*, most of which have the advantage of making use of a previously established infrastructure, remain above 1 m precision except for ultra-wideband based systems, which reach some tens of cm. Camera-based systems position below 5 cm, with high future potential due to rapid improvements in computer vision, having as their main disadvantages the relative expense of installing a multi-camera infrastructure and the need for very accurate calibration. Finally, ultrasound systems, based on time-of-flight measurements and perhaps the most comparable to the proposed system, reach precisions of below 2 cm.

It is always difficult to conduct an objective comparison of different technologies using different specifications, but two details should be noted when analyzing the proposed *IR* system: (A) Simplicity was taken as a design constraint, in order to achieve a cheap, modular and easy to extend and maintain solution; and (B) The precision achieved is strongly related to the photonic devices currently available on the market, and thus has the potential for improvement should new emitter and receiver devices with larger sensitive areas capable of working at higher power and frequency appear on the market. To illustrate this potential, note that using a 50% more powerful *LED* and a photodiode with twice the area and the same responsivity as the current ones, working at 20 MHz, the precision achieved would be more than 11 times greater (below 0.7 cm) using exactly the same measuring structure.

## 7. Conclusions and Future Research

An *IR*-based indoor robot locating method for Intelligent Spaces has been proposed as an alternative approach to other existing systems working with different technologies. The locating precision is below 10 cm, which falls within the precision achieved by the best existing systems, and is potentially improvable using the same measurement structure, should better performance photonic devices appear on the market. A simple, inexpensive and highly modular system has been developed so that covering a new area in a building is not complex, and enlarging an existing one would be an easy task.

The strong trade-off between coverage, precision and real time performance of the system has been solved by carefully designing the receiving and signal treatment stages. This renders it possible to recover phase information from very low quality signals, providing an effective *SNR* improvement of up to 37.5 dB. In addition, *I/Q* demodulation allows strong filtering at the output, which drastically reduces noise. Improved precision could be achieved by stronger filtering but at the cost of slower response, so that real time performance would be worse.

The main performance limitations are related to the trade-off between response time and maximum working power and sensitive area of the emitter and receiver devices, limiting *SNR* of received signals and maximum working frequencies, and consequently the final locating precision. It is important to note that this limitation is mainly related to the performance of current photonic devices rather than being a feature of the proposed locating method or system structure.

The current signal treatment stage is almost fully analog, centered on hardware *I/Q* demodulation. Implementing this stage on a digital system by applying AD-conversion immediately after reception is proposed as a future subject of research. This would increase robustness, repeatability and reproducibility of the system, as well as providing an easier test platform for new estimation algorithms.

Reducing the trade-off between coverage and precision is a key issue for positioning systems. To this end, an automatic orientation system for the receivers is proposed. This would increase the received power by effectively yielding higher responsivity in each sensor and would provide the possibility of using optics to increase the reception area. The orientation of receivers renders reception of power, and therefore *SNR* level, more independent of position. Implementing an auto-orientation system could potentially improve both precision and coverage of the *LPS*.

## Figures and Tables

**Table 1.** Different up-to-date commercial IR emitters (IRED).

**Device**	**P_max_(W)**	**I_max_(W/sr)**	**f_max_(MHz)**	**θ_1/2_(°)**	**λ_p_(nm)**	**I_Dmax_(DC, Pulsed) (A)**	**Evaluation**	
							**Positive**	**Negative**
SFH4050 (Osram)	0.5	70	23	80	850	100 mA, 1 A	Angle, Frequency	Power
OD-50W (OPC)	1	200	0.4	110	880	500 mA, 10A	Power Angle	Frequency
TSHG6200 (Vishay)	0.4	1800	14	10	850	100 mA, 1 A	Frequency	Angle
SFH4740 (10 leds) (Osram)	3.6	1800	28	60	850	1 A, 2 A	Power, Angle Frequency	Signal coherence
SFH4231 (Osram)	1	300	14	60	940	1 A, 2 A	Good trade-off solution	

**Table 2.** SNR levels in the output of the receiver.

**Measurement conditions (Distance and angle)**	**Amplitude**	**Noise (σ)**	**SNR**
2.5 m 0°	200 mV	150 mV	2.5 dB
3.5 m 45°	15 mV	150 mV	−20 dB
5 m 60°	5 mV	150 mV	−30 dB

**Table 3.** SNR levels in different stages after reception.

**Measuring conditions**	**Measuring point**	**Noise (σ)**	**SNR**
Distance = 2.5 m Angle = 0° Amplitude = 200 mV	Sensor output	150 mV	2.5 dB
	Demodulator output	7.5 mV	28.5 dB
	Digitized demodulator output	5 mV	32 dB
	Digital filter output	2 mV	40 dB
Distance = 3.5 m Angle = 45° Amplitude = 15 mV	Sensor output	150 mV	−20 dB
	Demodulator output	7.5 mV	6 dB
	Digitized demodulator output	5 mV	9.5 dB
	Digital filter output	2 mV	17.5 dB

**Table 4.** Comparison of indoor local positioning systems.

**Technology**	**Signal**	**Measure^1^**	**Positioning precision**
Bluetooth	RF	R.S.S.I.	2–3 m
RFID	RF	R.S.S.I.	1–2 m
Wi-Fi	RF	R.S.S.I.	1–2 m
GSM	RF	T.O.F.; R.T.O.F; D.T.O.F; A.O.A. R.S.S.I.	2–3 m
UWB	RF	T.O.F; R.T.O.F; D.T.O.F.	20–30 cm
Vision	Visible light	Pattern recognition	5 cm
Ultrasound	Ultrasound	T.O.F.; R.T.O.F; D.T.O.F.	1–2 cm
IR	Infrared	Differential phase-shift	7 cm
IR^2^	Infrared	Differential phase-shift	0.7 cm

**(1) T.O.F**.: Time of Flight **R.T.O.F**.: Round-trip Time of Flight **D.T.O.F**.: Differential Time of Flight **A.O.A**.: Angle of Arrival **R.S.S.I**.: Received Signal Strength Indicator.

**(2)** Estimated results: +50% emitted and received optical power. 20 MHz.
